# Electrochemical properties of MnSe inclusions and improving the pitting corrosion resistance of stainless steel via Se microalloying

**DOI:** 10.1038/s41598-024-57995-y

**Published:** 2024-03-26

**Authors:** Masashi Nishimoto, Tomoki Katsuyama, Izumi Muto

**Affiliations:** Department of Materials Science, Graduate School of Engineering, Tohoku University, 6-6-02 Aramaki Aza-Aoba, Aoba-ku, Sendai, 980-8579 Japan

**Keywords:** Metals and alloys, Corrosion

## Abstract

Controlling the electrochemical properties of non-metallic inclusions is of substantial interest in the design of corrosion-resistant alloys. To the best of our knowledge, the dissolution and pitting corrosion behavior of selenide inclusions in stainless steels and the improvement of the pitting corrosion resistance of type 304 stainless steels via Se microalloying have not been investigated. In this study, stainless steel specimens containing artificial MnS and MnSe inclusions were fabricated via spark plasma sintering to systematically investigate their electrochemical properties. The superior pitting corrosion resistance and dissolution resistance of MnSe to those of MnS were demonstrated. The results obtained from the sintered specimens were applied to improve the pitting corrosion resistance of type 304 stainless steels via Se microalloying. Adding a trace amount of Se (0.005 mass%) altered the readily soluble sulfide inclusions to dissolution-resistant selenide-type inclusions, resulting in improved pitting corrosion resistance of type 304 stainless steel.

## Introduction

Stainless steels play a key role in developing a sustainable society because they contribute to the durability and safety of steel structures owing to their superior corrosion resistance. However, pitting corrosion occurs in severely corrosive environments^1,2^. The pitting corrosion resistance of stainless steels can be improved by alloying with corrosion-resistant elements such as Ni and Mo, and highly alloyed stainless steels have been successfully used in severely corrosive environments^3–7^. However, considering the detrimental effects of using large amounts of alloying elements on the mechanical properties and excessive resource consumption, alternative methods for improving pitting corrosion resistance need to be developed.

Sulfide inclusions, such as MnS, are preferred initiation sites for pitting corrosion in stainless steels^8–12^. The presence of MnS inclusions on steel surfaces causes discontinuities in the passive films, leading to the chloride ions readily attacking these sites. However, recent studies have reported that even in commercial Type 304 stainless steels, exceptionally high pitting corrosion resistance in chloride solutions is exhibited at sites without MnS inclusions^13–19^. Thus, there is an increased possibility of dramatically improving the pitting corrosion resistance by rendering the MnS inclusions to be harmless without adding large amounts of alloying elements. This study focused on inclusion modification by adding trace amounts of alloying elements.

Pitting at MnS inclusions in stainless steels has been intensively studied, and anodic dissolution of MnS has been identified as the first step in the pit initiation process^20–31^. The dissolution of MnS exposes the bare surface of the steel matrix and produces S-species that are detrimental to the corrosion resistance of the steel matrix surrounding the inclusions, resulting in high pitting susceptibility near the inclusions in chloride solutions^32–34^. Therefore, preventing the dissolution of MnS is imperative to improving its pitting corrosion resistance. Further, the pitting corrosion resistance of stainless steels tends to improve as the inclusions become less soluble in chloride solutions^35–41^.

Selenium can improve the machinability of free-cutting steels by converting MnS into Mn(S,Se) inclusions^42,43^. Although a few studies mention the superior corrosion resistance of Se-added free-cutting steels to S-added free-cutting steels^43,44^, to the best of our knowledge, no systematic investigation has been conducted on the dissolution and pitting corrosion behavior of selenide inclusions in stainless steels. Furthermore, adding trace amounts of Se to improve the pitting corrosion resistance of commonly used low-S stainless steels is yet to be considered.

This study aims to compare the electrochemical properties of MnS and MnSe inclusions and explore the possibility of improving the pitting corrosion resistance of Type 304 stainless steels via Se microalloying. Owing to the complicated chemical compositions of the actual inclusions in commercial stainless steels, it is challenging to draw a comparison of the electrochemical properties of the inclusions. Therefore, in this study, spark plasma sintering (SPS) was used to fabricate specimens containing artificial inclusions^45–47^. This technique not only enables an unbiased comparison of the electrochemical properties of the inclusions but also allows for facile fabrication of the specimens. After confirming the beneficial properties of MnSe using the sintered specimens, the effect of Se on the pitting corrosion resistance of Type 304 stainless steels prepared via arc melting was analyzed.

## Methods

### Specimens

Stainless steel specimens containing artificial inclusions were prepared via SPS^45–47^. Gas-atomized Type 304 stainless steel powders (Kojundo Chemical Laboratory) were mixed with either MnS (99.9% purity, Sigma-Aldrich) or MnSe (99.9% purity, Kojundo Chemical Laboratory) powders. Table 1 lists the chemical compositions of the stainless steel powder. The particle sizes of the stainless steel, MnS, and MnSe powders were less than 105, 149, and 180 μm, respectively. The S and Se contents in the mixed powders used to prepare the specimens containing either MnS or MnSe were 0.07 and 0.17 mass%, respectively. The mixed powders were sintered at 1373 K for 1200 s under vacuum with a uniaxial pressure of 40 MPa in an SPS system (LABOX-110, Sinter Land). The sintered steel specimens fabricated from mixed Type 304 and MnS powders and mixed Type 304 and MnSe powders were referred to as Steel A and Steel B, respectively.

**Table 1:** Chemical compositions of gas-atomized powder and sheets of type 304 stainless steel (mass%).

	C	Si	Mn	P	S	Ni	Cr	Mo	Cu	Ti	Al	N	O	Se
Powder	0.01	0.22	0.17	0.012	0.003	11.1	19.0	0.01	0.01	0.001	0.002	–	–	–
Steel C	0.04	0.55	0.90	0.031	0.003	8.2	18.0	0.20	0.29	0.003	0.003	0.031	0.002	< 0.0002
Steel D	0.05	0.54	0.89	0.026	0.003	8.2	18.0	0.17	0.25	0.003	0.003	0.034	0.002	0.005

–, not analyzed.

To investigate the effect of Se microalloying on the pitting corrosion resistance of low-S stainless steels, Se-free and Se-containing Type 304 stainless steels were prepared via arc melting. The chemical compositions of the steels are listed in Table 1. The steels were hot rolled at 1523 K to achieve a 75% reduction ratio. The heat treatment was conducted at 1273 K for 20 h, followed by quenching in water. The Se-free and Se-containing specimens were denoted as Steel C and Steel D, respectively.

Before the electrochemical measurements were taken, the specimen surfaces were ground using SiC papers and polished using a 1-μm diamond paste. Finally, the samples were rinsed with ethanol.

### Electrochemical measurements

To analyze the electrochemical properties of artificial MnS and MnSe inclusions in Steel A and Steel B, potentiodynamic anodic polarization was conducted in naturally aerated 0.1 M NaCl (pH 5.5) at an ambient temperature of approximately 298 K. A microscale electrode area (approximately 100 μm × 100 μm) that included a single artificial inclusion was used. Details of the electrochemical setup have been published elsewhere^24,48–50^. A Pt wire counter electrode and a small Ag/AgCl (3.33 M KCl) reference electrode (0.206 V vs. standard hydrogen electrode (SHE) at 298 K) were used. All potentials reported herein refer to those of the SHE. The scan rate of the electrode potential was 20 mV min^–1^. Polarization was repeated twice on each specimen to ensure reliability.

To evaluate the pitting corrosion resistance of Steel C and Steel D, potentiodynamic anodic polarization was conducted in naturally aerated 0.1 M NaCl (pH 5.5) at 298 K. The electrode area was approximately 10 mm × 10 mm. In addition, the open circuit potential (OCP) was measured for 24 h in naturally aerated 0.1 M NaCl at 298 K. The pH values of the solution were 5.5 (unadjusted) and 3.5 (adjusted with 0.1 M HCl). The electrode area for the OCP measurements was approximately 10 mm × 10 mm.

### Observation and analysis

The specimen surfaces were observed using an optical microscope and field-emission scanning electron microscope (FE-SEM) equipped with an energy-dispersive X-ray spectroscopy (EDS) system. Secondary electron images and EDS maps were obtained at an accelerating voltage of 20 kV.

## Results and discussion

### Electrochemical properties of the artificial inclusions

**Figure 1:**
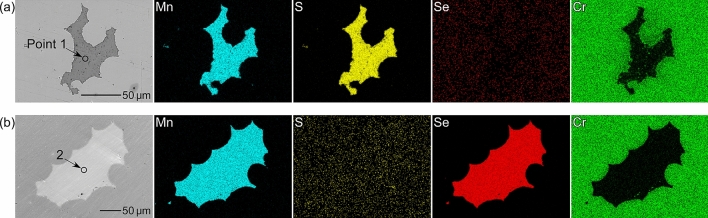
SEM images and EDS maps of the artificial inclusions in (**a**) Steel A and (**b**) Steel B.

**Figure 2:**
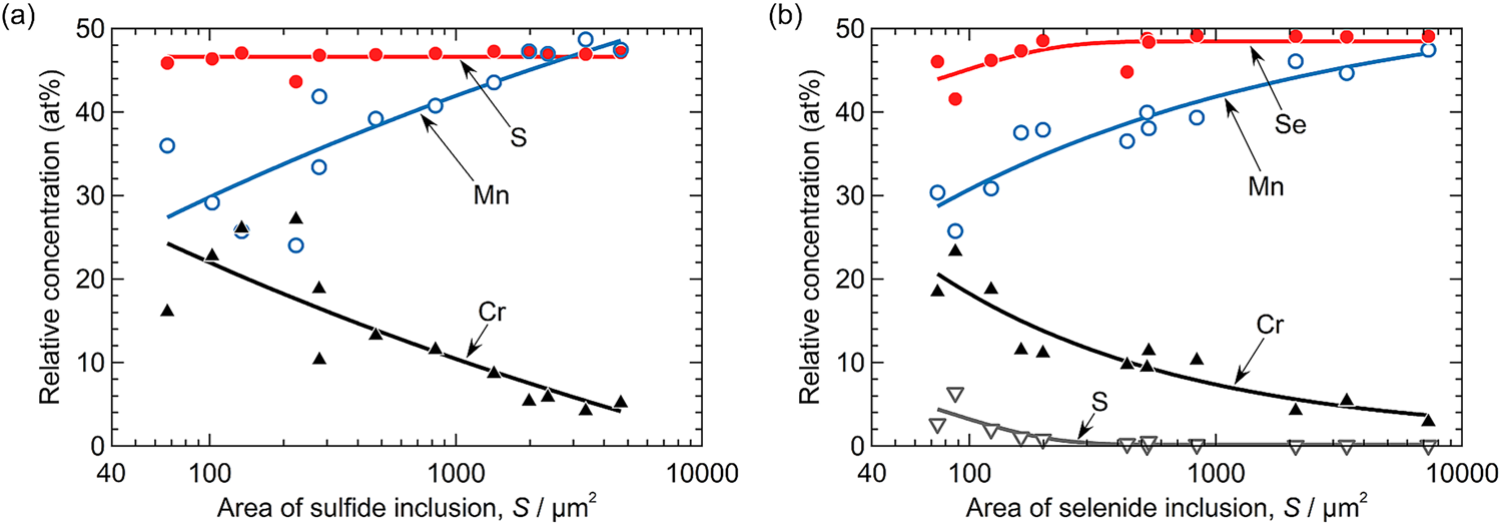
Relationship between the chemical composition and area of the artificial inclusions in (**a**) Steel A and (**b**) Steel B.

Figure 1 shows SEM images and EDS maps of the artificial inclusions in Steel A (sintered using mixed Type 304 and MnS powders) and Steel B (sintered using mixed Type 304 and MnSe powders). Table 2 presents the relative compositions at Points 1 and 2 in Fig. 1. As shown in Fig. 1a, b, the artificial inclusions in Steel A constitute Mn and S, whereas those in Steel B comprise Mn and Se. Both inclusions contain small amounts of Cr, but the purities of the raw MnS and MnSe powders are 99.9%. It is likely that Cr diffused from the stainless steel powders during the sintering process. These inclusions can be accurately written as (Mn,Cr)S and (Mn,Cr)Se, but for simplicity, they are referred to as MnS and MnSe, respectively. The Cr concentration differed for each artificial inclusion. Figure 2a, b show the relationship between the chemical composition and area of the artificial inclusions. For both specimens, the larger artificial inclusions tend to have lower Cr and higher Mn concentrations. In this study, the polarization behavior of the artificial inclusions containing 10 at% Cr was examined because the MnS inclusions in commercial Type 304 stainless steels contain approximately 10 at% Cr^24,51,52^. In the following experiments, the artificial inclusions that were examined were first characterized using EDS analysis. The specimen surfaces were briefly re-polished with a 1-μm diamond paste, and the polarization curves were measured. This procedure enables an unbiased comparison between the electrochemical properties of the artificial MnS and MnSe inclusions.

**Table 2:** Relative compositions (at%) at points 1 and 2 in Fig. 1.

	Mn	S	Se	Cr	O	Fe	Ni
Point 1	49	47	< 1	4	< 1	< 1	< 1
Point 2	48	< 1	49	2	< 1	< 1	< 1

**Figure 3:**
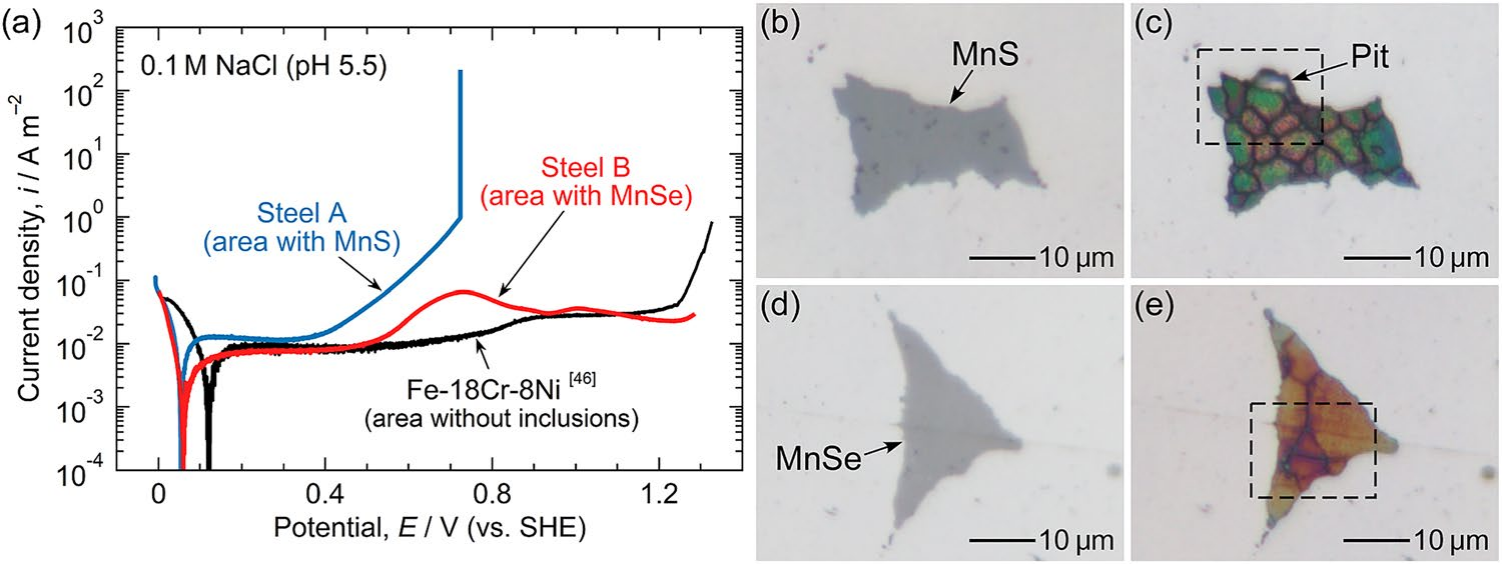
(**a**) Polarization curves of microscale electrode areas with a single artificial inclusion measured in 0.1 M NaCl (pH 5.5). The polarization curve of an area without inclusions on a Fe–18Cr–8Ni stainless steel is quoted from the literature^46^ (adapted under the terms of the CC BY 4.0 Creative Commons license, Copyright 2020, The Authors, published by Elsevier Ltd.). Optical micrographs of the artificial inclusions in (**b,c**) Steel A and (**d,e**) Steel B: (**b,d**) before and (**c,e**) after polarization.

Figure 3a shows the polarization curves of the microscale electrode areas with a single artificial inclusion. Figure 3b–e depict optical micrographs of the artificial inclusions before and after the polarization of Steel A and Steel B. The polarization curve of an area without inclusions on a Fe–18Cr–8Ni stainless steel is quoted from our previous research^46^. For all steels, polarization began at 0 V, and the cathodic currents flowed first. Anodic currents were measured above approximately 0.1 V. In the black curve (the area without inclusions), the passive state of the stainless steel was observed until 0.8 V. The slight increase in current density at approximately 0.8 V was attributed to the transpassive dissolution. An oxygen evolution reaction occurred above approximately 1.2 V. For the area with an artificial MnS inclusion (represented by the blue curve), the current density gradually increased above approximately 0.4 V. This gradual increase was attributed to the anodic dissolution of MnS. Many researchers have reported that MnS inclusions in Type 304 stainless steels start to dissolve above approximately 0.4 V (vs. SHE)^13,46,52,53^. At 0.72 V, a large increase in the current density is observed, indicating the initiation of pitting corrosion. As shown in Fig. 3b, the surface of MnS is gray before polarization. After polarization (Fig. 3c), the color of MnS changes, and a pit is observed at the boundary between MnS and the steel matrix. Discoloration of the inclusion indicates dissolution of the inclusion surface^24,29,39,46^. For the area with an artificial MnSe inclusion (represented by the red curve), the current density slightly increases at approximately 0.5 V. This increase was attributed to the anodic dissolution of the MnSe because the passive current density of the stainless steel matrix is approximately 1 × 10^–2^ A m^–2^. The current increase owing to the inclusion dissolution is smaller than that of Steel A. Moreover, no current spike is observed until 1.28 V. As shown in Fig. 3d, e, the color of the MnSe changes after polarization; however, no pits are generated. This suggests that the pitting corrosion resistance of MnSe is better than that of MnS.

**Figure 4:**
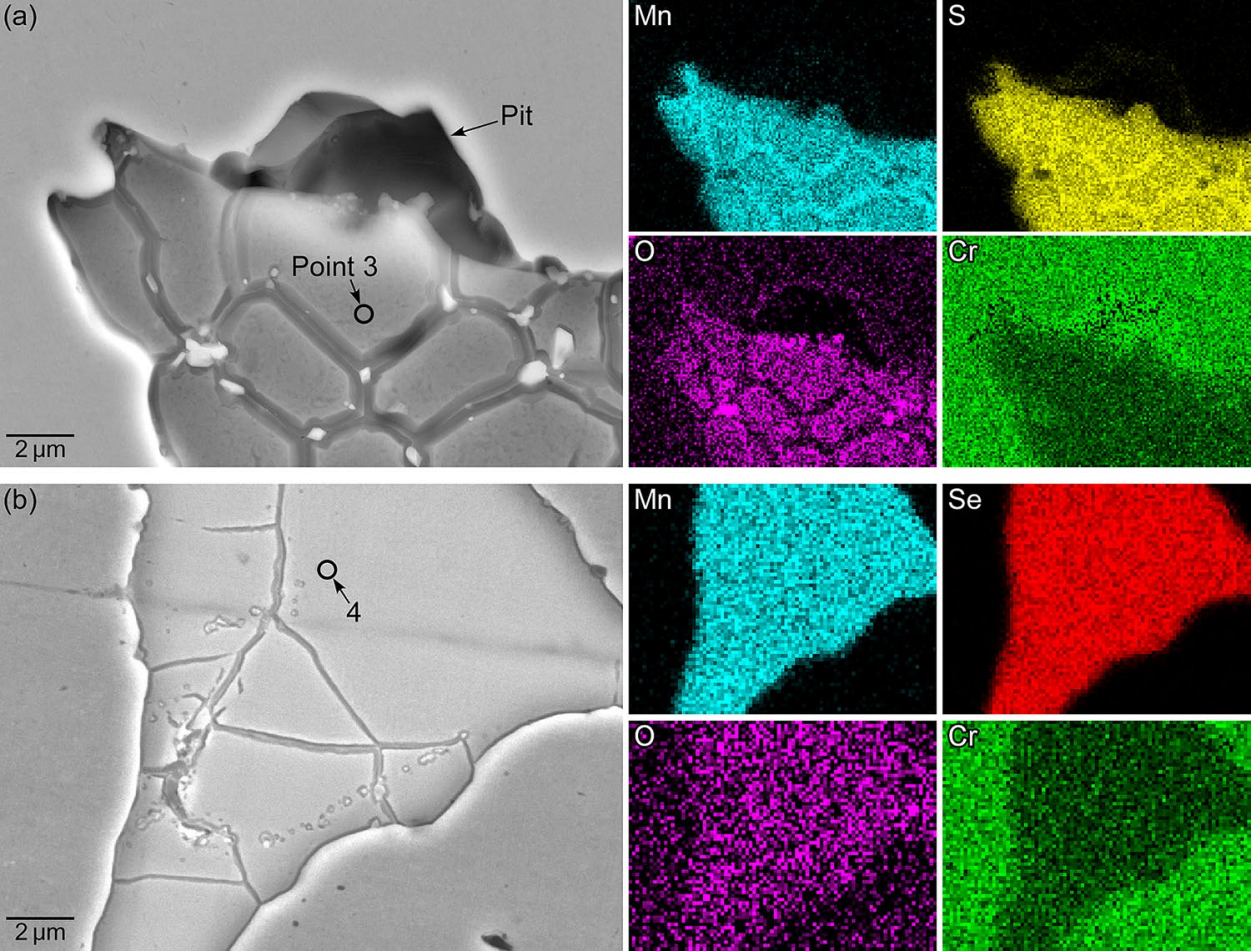
SEM images and EDS maps of the areas surrounded by the dashed lines in Fig. 3c, e: (**a**) Steel A and (**b**) Steel B.

Figure 4a, b show SEM images and EDS maps of the areas surrounded by the dashed lines in Fig. 3c, e. The surfaces of both inclusions dissolved slightly; however, compared to MnS, minor dissolution of MnSe was observed even though it was polarized at higher potentials. For MnS, the S concentration decreased from 47 to 35 at%, and O was detected. For MnSe, the Se concentration decreased slightly to 44 at%. This suggests that MnSe exhibits superior dissolution resistance to MnS. Table 3 presents the relative compositions (at%) of the artificial inclusions before and after polarization.

**Table 3:** Relative compositions (at%) of the artificial inclusions in Fig. 4 before and after polarization.

	Mn	S	Se	Cr	O	Fe	Ni
As-polished MnS	42	47	< 1	10	< 1	< 1	< 1
Point 3 (after polarization)	29	35	< 1	10	25	< 1	< 1
As-polished MnSe	39	< 1	49	11	< 1	< 1	< 1
Point 4 (after polarization)	31	< 1	44	9	15	< 1	< 1

The MnSe inclusions initially acted as an anodic site. As shown in Fig. 3a, the increase in anodic current density was observed on Steel B in the potential range of 0.5 to 0.9 V. In Fig. 4b, the surface of the MnSe slightly dissolved, while no corrosion damage was observed on the stainless steel matrix surrounding the MnSe. However, whether MnSe inclusions continue acting as an anodic site during the whole pitting corrosion process is unclear. In the case of the MnS inclusions in Type 304 stainless steel, it has been proposed using a finite element method model that the MnS inclusions transform from the anodic to cathodic sites during the pit initiation process^28^. Further explorations are necessary to elucidate the pitting corrosion behavior at selenide inclusions.

To consider the dissolution resistance of the inclusions from a thermodynamic point of view, the solubility products of MnS and MnSe were calculated using the Gibbs energies of formation obtained from the HSC thermochemical database and literature^54,55^. The reaction of manganese chalcogenide (MnX) dissolving in water to produce divalent manganese ions (Mn^2+^) and chalcogenide ions (X^2–^) is given by:

1 $${\text{MnX}}\left({\text{s}}\right)\rightleftharpoons {{\text{Mn}}}^{2+}\left({\text{aq}}\right)+{{\text{X}}}^{2-}\left({\text{aq}}\right)$$

The solubility product, *K*_sp_, for MnX is calculated using the following equations:

2 $${K}_{{\text{sp}}}=\left[{{\text{Mn}}}^{2+}\right]\left[{{\text{X}}}^{2-}\right]$$

3 $${\Delta G}^{^\circ }=-RT{\text{ln}}{K}_{{\text{sp}}}$$

where Δ*G*^◦^ is the Gibbs energy change of the reaction (J mol^–1^), *R* is the gas constant (J K^–1^ mol^–1^), and *T* is the temperature (K). Table 4 shows the calculated solubility products of MnS and MnSe at 298 K. The solubility product of MnSe is lower than that of MnS, suggesting that MnSe is less soluble than MnS.

**Table 4:** Solubility products of chalcogenides and the standard chemical potentials of manganese ions, chalcogenide ions, and chalcogenides at 298 K^54,55^.

	${K}_{\text{sp}}$ (mol^2^ L^–2^)	${\mu }_{{\text{Mn}}^{2+}}^{^\circ }$ (kJ mol^–1^)	${\mu }_{{\text{X}}^{{2}-}}^{^\circ }$ (kJ mol^–1^)	${\mu }_{{\text{MnX}}}^{^\circ }$ (kJ mol^–1^)
MnS	4.181 × 10^–14^	–227.997	85.973	–218.347
MnSe	5.440 × 10^–20^	–227.997	178.238	–159.659

**Figure 5:**
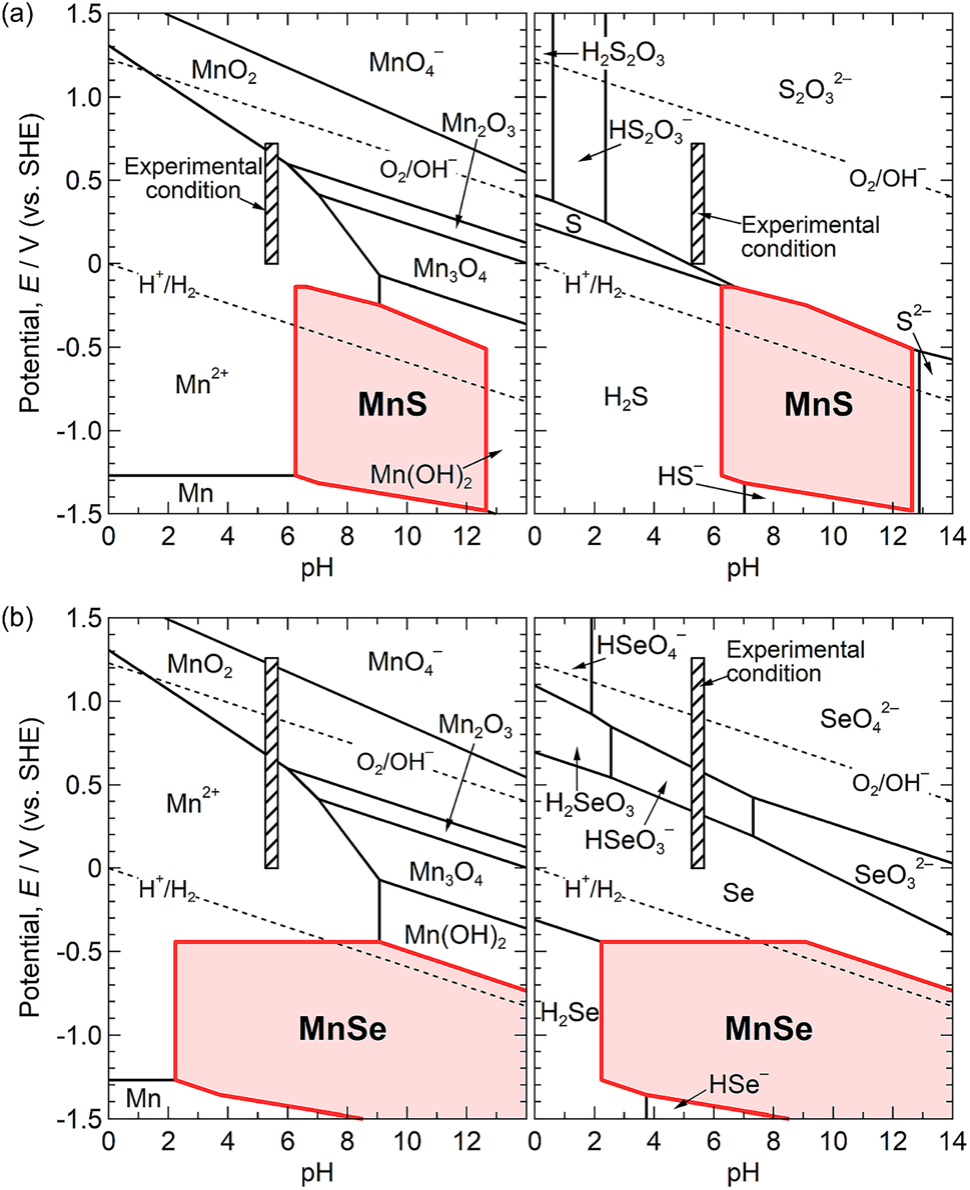
Potential-pH diagrams for (**a**) Mn–S–MnS–H_2_O and (**b**) Mn–Se–MnSe–H_2_O systems at 298 K. The standard chemical potentials of Mn-species, S-species, MnS, and MnSe were obtained from the HSC thermochemical database^54^, and those of Se-species were obtained from the literature^55^. The concentration of soluble species is 1.0 × 10^−3^ mol kg^−1^ (H_2_O). The slashed rectangles indicate the experimental conditions of the polarization curves shown in Fig. 3a.

The potential-pH diagrams were also calculated to consider the superior dissolution resistance of MnSe in terms of pH and electrode potential. Figure 5 shows the potential-pH diagrams of the Mn-S-MnS-H_2_O and Mn-Se-MnSe-H_2_O systems at 298 K. The standard chemical potentials of Mn-species, S-species, MnS, and MnSe were obtained from the HSC thermochemical database^54^, and those of Se-species were obtained from the literature^55^. Thiosulfate ions were assumed to be released from the dissolution of sulfides^32,33^. The stable regions of MnS and MnSe were located at low potentials. Although the pH range of the stable region for MnS was higher than 6, MnSe remained stable even at approximately pH 3. The thermodynamic stability of MnSe is expected to prevent the dissolution of inclusions in near-neutral to weakly acidic solutions. In addition, a large stable region of Se was located above the MnSe region. This implies that Se remains on MnSe inclusions even if a preferential dissolution of Mn from the inclusions occurs. The properties of the inclusion surfaces may also be related to the superior dissolution resistance of MnSe inclusions; however, the details remain unclear and should be explored in future research.

### Improved pitting corrosion resistance of Type 304 stainless steel via Se microalloying

**Figure 6:**
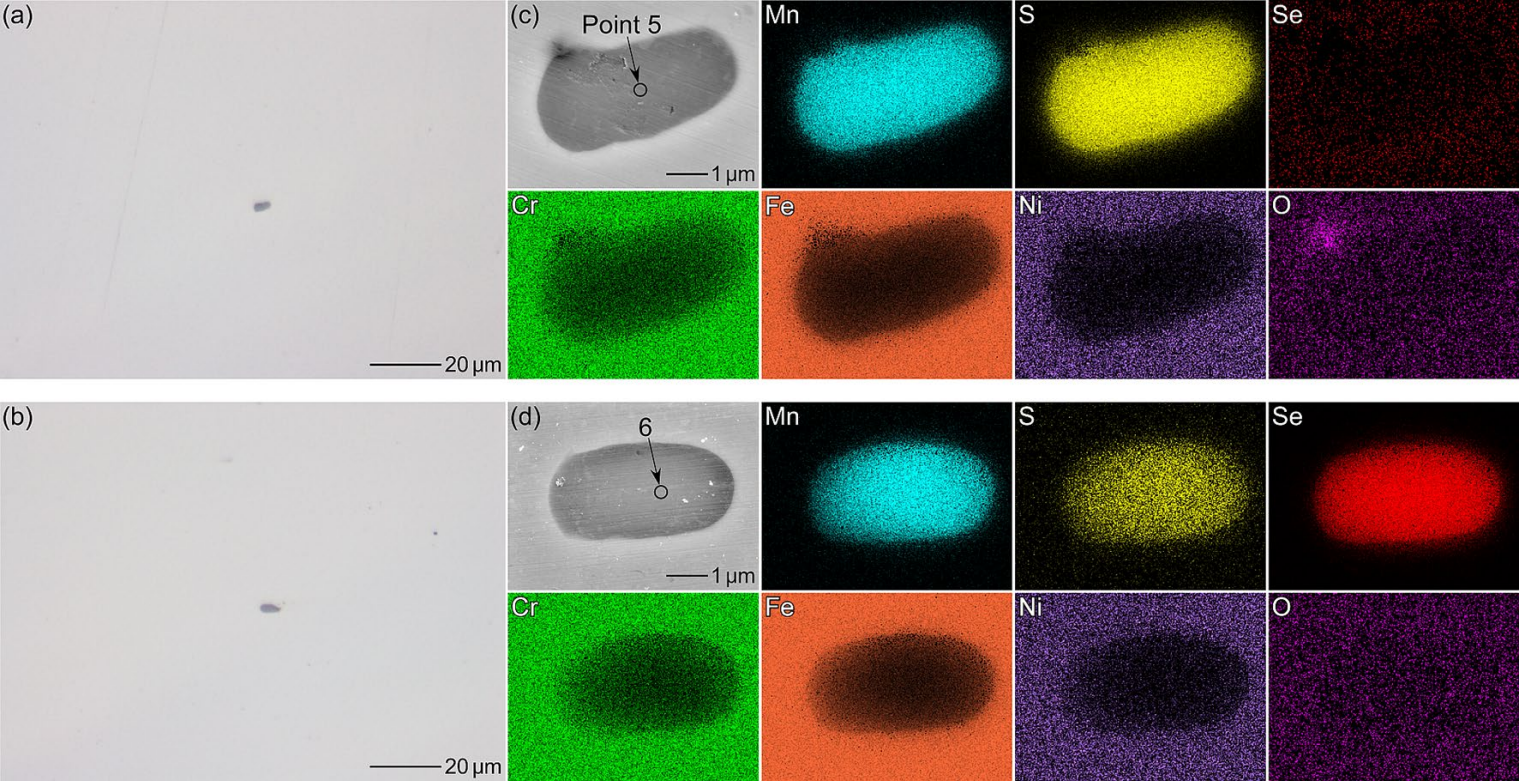
Optical micrographs of the as-polished surfaces of (**a**) Steel C and (**b**) Steel D. SEM images and EDS maps of the inclusions in (**c**) Steel C and (**d**) Steel D.

To utilize the electrochemical properties of MnSe for improving the pitting corrosion resistance of stainless steels, a Type 304 stainless steel specimen with a trace amount of Se (0.005 mass%) was fabricated via arc melting. Figure 6 shows optical micrographs, SEM images, and EDS maps of Steel C (Se-free) and Steel D (Se-added). Table 5 presents the results of the EDS analysis at Points 5 and 6. The small gray particles in Fig. 6a, b represent non-metallic inclusions. For both steels, the size of the inclusions was less than approximately 5 µm in diameter, and the distribution density was approximately 50 inclusions/mm^2^. In Steel C, the inclusions were mainly composed of Mn and S. In contrast, Mn, S, and Se were detected in the inclusions of Steel D. As shown in Fig. 6d, S and Se existed uniformly in the inclusions. More results of the EDS analysis of the inclusions can be found as Supplementary Fig. S1 and Supplementary Table S1 online. These results suggest that the sulfide inclusions were modified into selenide-type inclusions via Se microalloying.

**Table 5:** Relative compositions (at%) at points 5 and 6 in Fig. 6.

	Mn	S	Se	Cr	O	Fe	Ni
Point 5	38	35	< 1	9	< 1	16	2
Point 6	34	8	33	7	< 1	16	2

**Figure 7:**
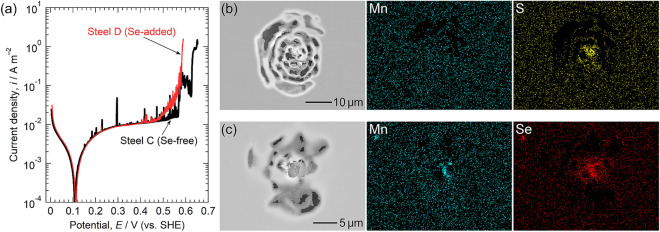
(**a**) Polarization curves of Steel C and Steel D measured in 0.1 M NaCl (pH 5.5). The electrode area was approximately 10 mm × 10 mm. SEM images and EDS maps of pits initiated on (**b**) Steel C and (**c**) Steel D.

The effect of Se microalloying on the pitting corrosion resistance was first analyzed via potentiodynamic polarization in 0.1 M NaCl (pH 5.5). Figure 7a shows the polarization curves of Steel C and Steel D. For Steel C, many current spikes due to metastable pitting were observed from approximately 0.1 to 0.5 V. The sudden large increase in current density at 0.57 V implies the initiation of stable pitting. For Steel D, few current spikes were observed until 0.4 V, indicating that metastable pitting events decreased via Se microalloying as expected. However, the current density gradually increased above approximately 0.5 V due to the anodic dissolution of the selenide inclusions. A sudden increase in current density at 0.58 V is indicative of the initiation of stable pitting. The improvement in the pitting potential of Steel D was not as significant as expected. Figure 7b, c depict SEM images and EDS maps of the pits observed after polarization. Pits with lacy covers were initiated on both specimens. The signals of S and Se near the center of the pits indicate that sulfide and selenide-type inclusions were the initiation sites of pitting on Steel C and Steel D, respectively.

Pitting potential is likely unsuitable for the comparison of the pitting corrosion resistance of Steel C and Steel D. Although the sulfide inclusions were modified into selenide-type via Se microalloying, these selenide-type inclusions still contain some amounts of S. As evident in Fig. 3, the anodic dissolution of MnSe occurs above approximately 0.5 V, and thus, pitting corrosion at selenide-type inclusions is inevitable above 0.5 V during anodic polarization. In Fig. 7a, the current density of Steel D started to increase at approximately 0.5 V, which is well correlated with the result in Fig. 3a. The anodic dissolution of MnSe above 0.5 V may be attributed to the breakdown of the surface film on MnSe. Tokuda et al*.* fabricated a bulk (Mn,Cr,Fe)S specimen and analyzed its electrochemical property^56^. They revealed that three regions appear in the polarization curve of the (Mn,Cr,Fe)S: the active dissolution region of (Mn,Cr,Fe)S around –0.2 V (vs. SHE), the region of the oxide film formation on the (Mn,Cr,Fe)S surface from 0.2 to 0.5 V, and the region of the breakdown of the surface oxide film above 0.5 V. Similar polarization behavior has been found on actual MnS inclusions in Type 304 stainless steel using a microcapillary electrochemical cell^14^. Therefore, it is considered that the pitting corrosion resistance of Steel D should be assessed at lower potentials below the anodic dissolution potential of MnSe.

**Figure 8:**
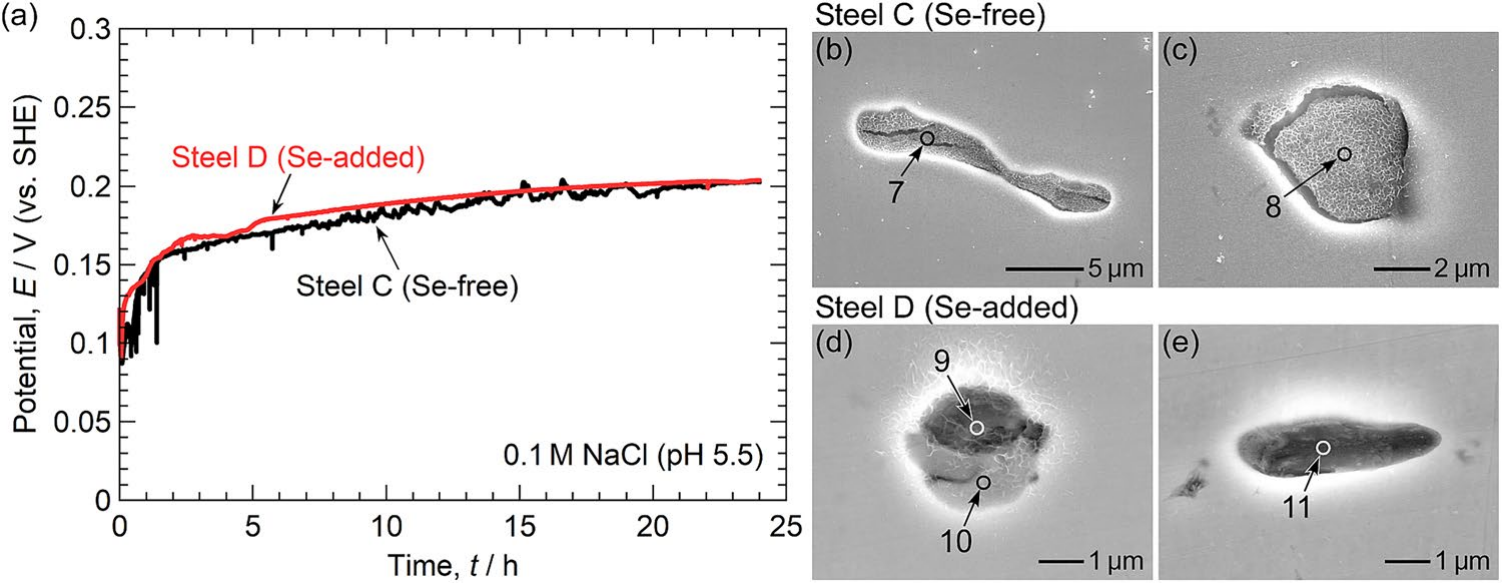
(**a**) Temporal variation of the open circuit potential of Steel C and Steel D during immersion in 0.1 M NaCl (pH 5.5). The electrode area was approximately 10 mm × 10 mm. SEM images of the inclusions captured after the immersion tests: (**b,c**) Steel C and (**d,e**) Steel D.

Based on the above results, immersion tests are appropriate for analyzing the effect of Se microalloying on the pitting corrosion resistance rather than potentiodynamic polarization to higher potentials. Figure 8a shows the temporal variation in the OCPs of Steel C and Steel D measured in 0.1 M NaCl (pH 5.5). For both steels, the OCPs gradually increased to 0.2 V, which is approximately 0.3 V lower than the anodic dissolution potential of MnSe. For Steel C, sudden large drops of the OCP occurred several times within the first 2 h of immersion, and the oscillation of the OCP was observed during the 24 h immersion period. The reductions and oscillation of the OCP were likely due to pitting events. In contrast, no reductions and oscillation of the OCP were observed for Steel D. This indicates that the pitting corrosion resistance of Steel D (Se-added) was higher than that of Steel C (Se-free). Figure 8b–e depict SEM images of the inclusions captured after immersion. Table 6 presents the results of the EDS analysis at Points 7–11. Slight dissolution of the inclusions was observed for both steels, but the inclusion dissolution appears to be more minor in Steel D. For the inclusions in Steel C, little or no S signal was detected in the EDS analysis. On the other hand, S and Se remained in the inclusions in Steel D. The results suggest that the dissolution of the inclusions was inhibited by the inclusion modification from sulfide to selenide-type, resulting in improved pitting corrosion resistance of Type 304 stainless steel via Se microalloying.

**Table 6:** Relative compositions (at%) at points 7–11 in Fig. 8.

	Mn	S	Se	Cr	O	Fe	Ni	Al	Mg	Ti
Point 7	< 1	< 1	< 1	23	15	57	5	< 1	< 1	< 1
Point 8	< 1	< 1	< 1	19	20	53	7	< 1	< 1	< 1
Point 9	< 1	< 1	2	15	21	48	4	3	6	< 1
Point 10	< 1	4	17	11	20	36	4	5	2	< 1
Point 11	< 1	3	33	14	5	39	4	1	< 1	< 1

**Figure 9:**
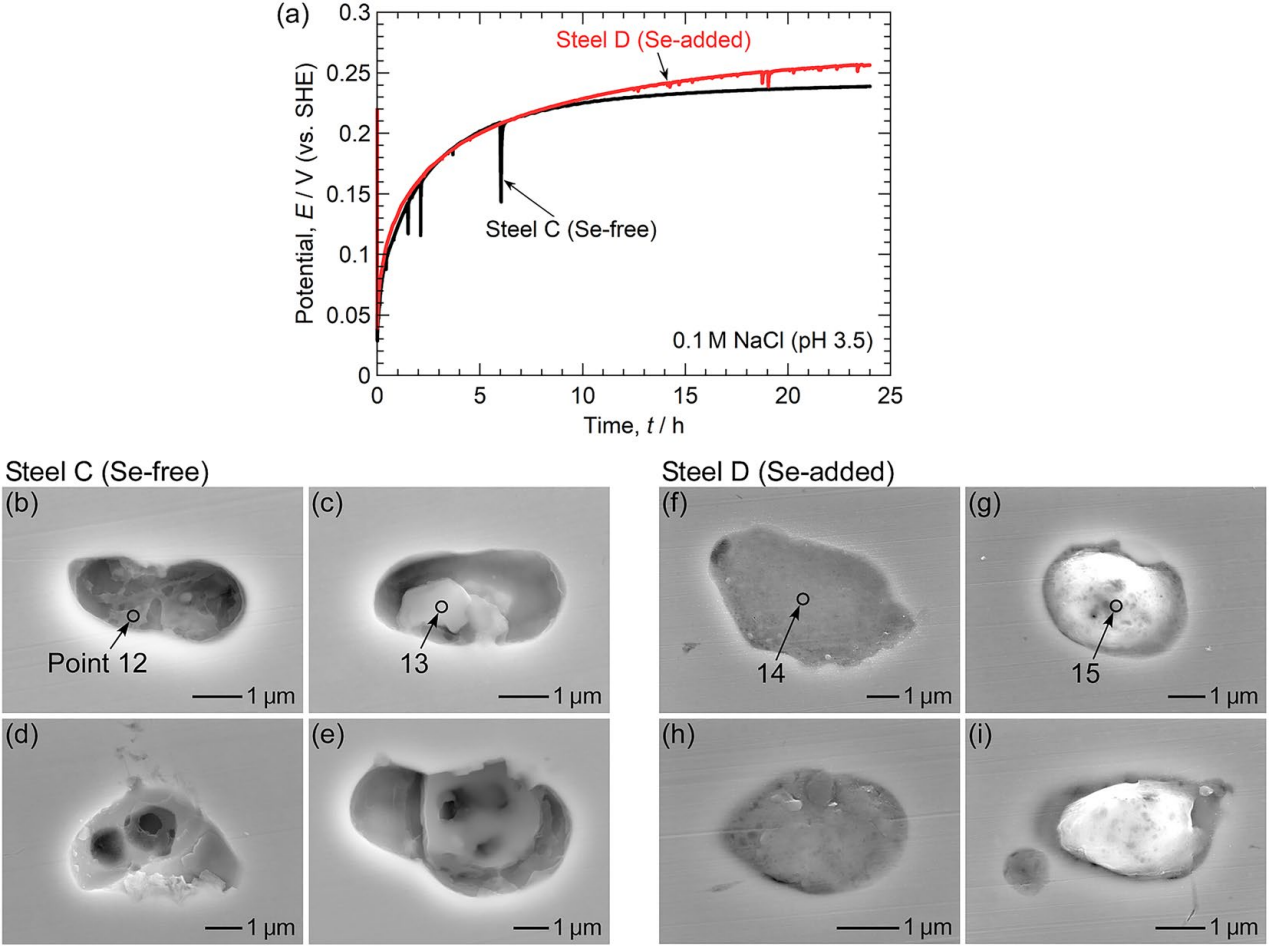
(**a**) Temporal variation of the open circuit potential of Steel C and Steel D during immersion in 0.1 M NaCl (pH 3.5, adjusted with 0.1 M HCl). The electrode area was approximately 10 mm × 10 mm. SEM images of the inclusions captured after the immersion tests: (**b–e**) Steel C and (**f–i**) Steel D.

The potential-pH diagrams in Fig. 5 imply that the dissolution resistance of MnSe is high even in weakly acidic solutions. To verify this speculation, immersion tests were also conducted in 0.1 M NaCl (pH 3.5, adjusted with 0.1 M HCl). Figure 9a shows the temporal variation in the OCPs of Steel C and Steel D. The OCPs gradually increased to approximately 0.25 V. The OCP of Steel C dropped several times within the first 6 h of immersion. In contrast, no OCP drop was observed until 12 h for Steel D. From 12 to 24 h, the drops of the OCP occurred, but these reductions were considerably smaller than those for Steel C. Figure 9b–i depict SEM images of the inclusions captured after immersion. Table 7 presents the results of the EDS analysis at Points 12–15. The inclusions in Steel C dissolved significantly (Fig. 9b–e), and little or no Mn and S signals were detected in the EDS analysis. Some of the residuals were oxides. However, most of the inclusions in Steel D remained undissolved. The Se concentration remained high even after 24 h of immersion. As shown in Fig. 9g, i, the inclusion surfaces appeared bright owing to charging, implying the formation of a low-conductivity layer on their surfaces. The inclusion surfaces were likely enriched in Se. The beneficial effect of Se microalloying on the dissolution resistance of the inclusions and the pitting corrosion resistance of Type 304 stainless steel was demonstrated not just in near-neutral pH solutions but also in weakly acidic solutions.

**Table 7:** Relative compositions (at%) at points 12–15 in Fig. 9.

	Mn	S	Se	Cr	O	Fe	Ni	Al	Mg	Ti
Point 12	1	1	< 1	21	10	60	7	< 1	< 1	< 1
Point 13	< 1	< 1	< 1	10	33	33	4	18	1	1
Point 14	1	2	34	14	6	37	5	< 1	< 1	< 1
Point 15	7	4	37	12	3	32	4	< 1	< 1	< 1

## Conclusions

In summary, we fabricated stainless steel specimens containing artificial MnS and MnSe inclusions via SPS to compare their electrochemical properties. Both artificial inclusions contained small amounts of Cr. The larger artificial inclusions tended to have lower Cr and higher Mn concentrations.

We demonstrated the superior pitting corrosion and dissolution resistances of MnSe to those of MnS. The electrochemical properties of the artificial inclusions were analyzed by potentiodynamic polarization of microscale electrode areas with either MnS or MnSe measured in 0.1 M NaCl. No pit was initiated at the MnSe, while a stable pit was generated at the MnS. The surfaces of both artificial inclusions dissolved slightly; however, minor dissolution of MnSe was observed compared to MnS. Thermodynamic calculation showed that MnSe is less soluble than MnS.

The findings obtained using the sintered specimens were applied to improve the pitting corrosion resistance of stainless steels prepared by arc melting. Adding a trace amount of Se (0.005 mass%) altered the readily soluble sulfide inclusions to dissolution-resistant selenide-type inclusions. The results of the immersion tests indicated that the improvement of the pitting corrosion resistance of Type 304 stainless steel via Se microalloying was demonstrated not just in near-neutral pH solutions but also in weakly acidic solutions.

### Supplementary Information

Supplementary Information.

## Data Availability

The datasets generated and/or analyzed during the current study are available from the corresponding author on reasonable request.
