# Supplementary material for: Electrochemical properties of MnSe inclusions and improving the pitting corrosion resistance of stainless steel via Se microalloying
Source: Sci Rep. 2024 Mar 26;14:7156. doi: 10.1038/s41598-024-57995-y (PMC10966076; doi:10.1038/s41598-024-57995-y)
Supplement: Supplementary file 1 — Supplementary Information. [file 41598_2024_57995_MOESM1_ESM.pdf]

Supplementary Information

Electrochemical properties of MnSe inclusions and improving the pitting corrosion resistance of stainless steel via Se microalloying

Masashi Nishimoto [*], Tomoki Katsuyama [*], and Izumi Muto

Department of Materials Science, Graduate School of Engineering, Tohoku University, 6-6-02 Aramaki aza-aoba, Aoba-ku, Sendai, 980-8579, Japan

[*] Corresponding authors:

masashi.nishimoto.b8@tohoku.ac.jp (M. Nishimoto)

tomoki.katsuyama.p5@dc.tohoku.ac.jp (T. Katsuyama)

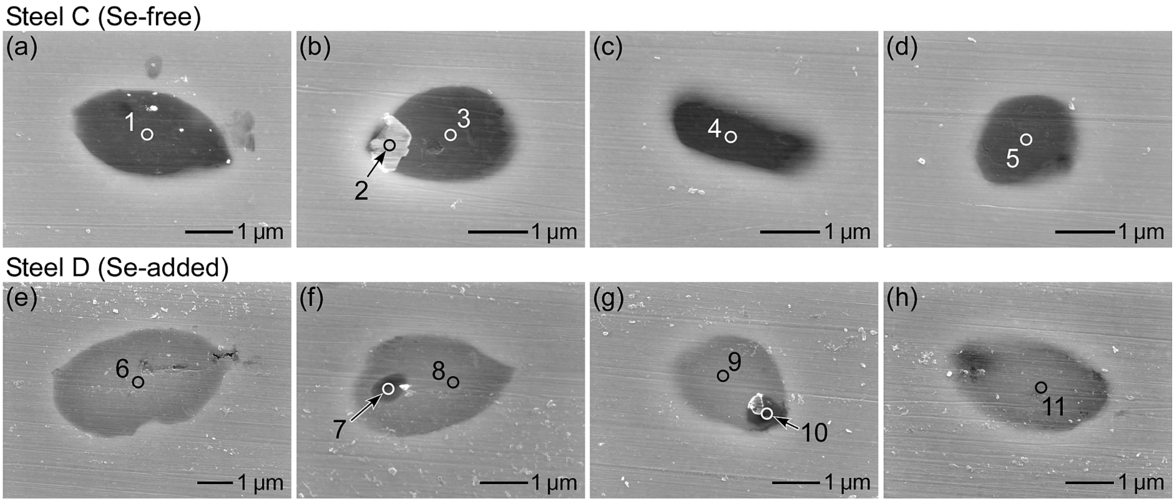

Supplementary Fig. S1 SEM images of the inclusions in (a–d) Steel C and (e–h) Steel D.

Supplementary Table S1 Relative compositions (at%) at Points 1–11 in Supplementary Fig. S1.

	Mn	S	Se	Cr	Fe	Ni	O	Al	Mg	Ti
Point 1	31	29	<1	11	26	2	<1	<1	<1	<1
Point 2	6	5	<1	10	29	3	29	16	2	<1
Point 3	25	24	<1	13	34	3	<1	<1	<1	<1
Point 4	34	27	<1	11	25	3	<1	<1	<1	<1
Point 5	29	25	<1	13	30	3	<1	<1	<1	<1
Point 6	25	2	35	10	25	3	<1	<1	<1	<1
Point 7	17	1	18	9	21	2	22	3	<1	7
Point 8	27	2	33	10	25	3	<1	<1	<1	<1
Point 9	35	2	35	7	16	2	2	1	<1	<1
Point 10	11	<1	9	11	26	3	26	8	<1	6
Point 11	40	3	37	6	13	1	<1	<1	<1	<1
